# Exploring Trauma Simulation: Insights from the Simulation in Trauma Across Resident Training (START) Study and Trainee Experiences

**DOI:** 10.7759/cureus.72502

**Published:** 2024-10-27

**Authors:** Jeremy Chan, James Barnett, Charmilie Chandrakumar, Amit Zaveri, Jamie McConnell, Kapil Sugand, Akash Patel

**Affiliations:** 1 General Medicine, Foundation Programme, Colchester Hospital, Colchester, GBR; 2 Trauma and Orthopedics, Royal National Orthopedic Hospital, London, GBR; 3 Trauma and Orthopedics, Royal Free London NHS Foundation Trust, London, GBR

**Keywords:** covid-19, orthopedic surgeons, orthopedic surgery, simulation training, virtual reality

## Abstract

Introduction and objective

Advancements in technology have presented an opportunity to incorporate simulation training into the trauma and orthopedic (T&O) curriculum. This study aimed to assess the perspective of T&O trainees regarding the acceptability and perceived educational impact of simulation training before and after a training day. This includes identifying the resources that trainees are likely to use and find helpful when preparing for a new or unfamiliar procedure, as well as evaluating their opinions on different levels of simulation fidelity. The null hypothesis for this study is that there is no difference in educational value after exposure to various simulation modalities.

Methods

This was a three-arm crossover cohort observational study involving 18 T&O specialty trainees in their first three years of training who attended a simulation training course, involving four independent stations with simulation tasks of varying fidelities. Pre-course and post-course questionnaires with a 10-point Likert scale to determine if simulation training exposure affected the perception of educational value. Data were treated non-parametrically with median (±median absolute deviation; 95% Bonnett-Price CI). Statistical significance was calculated using the Mann-Whitney U test and set as p<0.05.

Results

Twelve trainees (67%) had not previously used orthopedic simulators to prepare for unfamiliar operations. Ninety-four percent of trainees thought that simulation training could support safe practice, and all trainees considered simulation to be useful for training. Trainees strongly emphasized the importance of simulation training (pre-course vs. post-course median score: 7 vs. 9, p=0.01), the necessity for it to be readily assessable (9 vs. 10, p=0.061), its role in formal assessment (4 vs. 8, p=0.006), and being able to become a better surgeon from it (7 vs. 8, p=0.078).

Conclusions

T&O trainees acknowledged the value of simulation training in complementing their operating numbers; however, their access to these resources was limited - a problem well recognized in current literature. The gap in the implementation of simulation training should be addressed for T&O trainees to benefit from these resources fully.

## Introduction

Simulation trauma and orthopedic training

Trauma and orthopedic (T&O) specialty training has historically followed an apprenticeship-style approach as follows: trainees learn surgical skills by assisting senior colleagues, gradually progressing to perform procedures with increasing levels of autonomy while rotating through different subspecialties. However, technological advances presented an opportunity to incorporate simulation training into the curriculum, complementing traditional teaching methods [1]. Simulation provides a safe environment for trainees to practice techniques under close supervision, without risking patient safety. The emergence of the COVID-19 pandemic in 2019 reduced surgical cases across all specialties globally, including trauma and orthopedics (T&O), making it more challenging for trainees to acquire the necessary skills to operate proficiently [2]. Additional challenges include overcoming the long learning curves of arthroscopic procedures and increased consultant-led services [3-8]. The use of cadaveric models is increasingly discouraged due to ethical and financial issues [9-11]. The medicolegal argument for increased simulation practice is also compelling, as surgical complications may expose doctors and hospitals to liability.

Simulation modalities

Simulation training offers a potential solution to these challenges and promotes skill acquisition by providing training outside operating theatres and wet laboratories. Various modalities include online resources, textbooks, journals, and manufacturers' operative technique guides. Digital applications like Touch Surgery (London, UK: Digital Surgery LTD) demonstrated valid evidence and are easily accessible adjuncts to surgical education [12-15]. Low-fidelity "Box trainer" models are regularly used in laparoscopic surgical training, but their utility in T&O training remains unproven [16]. Virtual reality (VR) simulators, such as ARTHRO Mentor (Littleton, CO: 3D Systems) and ArthroS (Zürich, Switzerland: VirtaMed AG), offer high face validity and realistically simulate arthroscopic procedures [17]. Artificial Sawbones provide a realistic simulation of human bone and are widely used in simulation laboratories for training [18]. The continued development of high-fidelity technologies provides an opportunity to bridge the gap faced by T&O trainees.

Formal implementation solutions for simulation

Simulation training can be introduced through readily accessible simulators or formally organized simulation days. The former allows for regular practice, while the latter enables directed tuition from experienced trainers on the day [19]. Although the implementation of medical simulation training in the United Kingdom and Europe is present yet carried out variably, the utility and importance of simulation training are recognized, expected to grow, and has become mandatory in training [20-22].

## Materials and methods

In this three-arm crossover cohort observational study, the regional training day was held in November 2021 in a dedicated educational center within the T&O department of a single hospital. Eighteen participants rotated through four independent stations convened by senior orthopedic fellows and attendings with simulation tasks. These tasks of varying fidelities included the following: high-fidelity VR total knee replacement and femoral nailing (Selzach, Switzerland: Synthes Holding AG), high-fidelity VR femoral nailing (Selzach, Switzerland: Synthes Holding AG), medium-fidelity Sawbones femoral nailing (Vashon, WA: Pacific Research Laboratories), and low-fidelity: Fundamentals of Arthroscopic Surgery Training Program [20].

The inclusion criteria were T&O specialty trainees in their first three years of training (postgraduate T&O trainees years one to three). Any participant more junior or senior or who did not complete the pre- and post-questionnaire after attending the simulation day was excluded from the study. The research team compiled a questionnaire administered both pre- and post-course, validated using a modified Delphi technique by three independent T&O registrars. After three rounds, a final version was achieved by consensus.

A pre-course questionnaire was sent electronically to all participants one week prior. Participants were mandated to attend as part of their formal educational program to avoid selection bias. The pre-course questionnaire inquired about participants' demographics, experiences, and opinions before attending the course. Questions covered their training level, perceived gaps in training exacerbated directly by COVID-19, preparations for a new or unfamiliar operation, and the importance of surgical simulation. The majority of the questions allowed participants to choose one or more answers based on their opinions. Some questions were statements to which participants responded on a 10-point Likert scale. A score range of 1-3, 4-6, and 7-10 indicated weak, moderate, and strong agreement with the statements in the questionnaire, respectively. We collected responses between July and September 2021.

Immediately after the course, all participants were invited to complete a post-course questionnaire comprising of the pre-course questionnaire alongside their views on each station, their educational value, acceptability, and the importance of both surgical and orthopedic simulation (refer to appendix 1 for both the pre- and post-course questionnaires).

Only participants who completed both questionnaires and attended the training day course were included in the final analysis. We used simple descriptive analyses to describe the number of participants and percentages. Since the data were non-parametric, the median, median absolute deviation, and Bonnet-Price 95% confidence interval were calculated. Mann-Whitney U test calculated p-values, which were set at p<0.05. Box and Whisker Plot diagrams were compiled to demonstrate ranges. All data were treated using Microsoft Excel (Redmond, WA: Microsoft Corp.) and SPSS Statistics, version 28.0 and 29.0 (Chicago, IL: SPSS Inc.), and we tabulated data using Numbers (Cupertino, CA: Apple Inc.).

All participants consented for their data to be utilized for research purposes. All data were anonymized. No ethical approval was required since this reflected the service provision of the educational requirements of postgraduate trainees as part of the formal curricular program. The results were used as feedback to improve subsequent simulation days. In accordance with the Statement of Human and Animal Rights, no humans or animals were brought to harm in this study.

## Results

Demographics

All 18 eligible participants completed both pre-course and post-course questionnaires, meeting our inclusion criteria without any dropouts. Demographic data are summarized in Table 1. Five (28%) trainees were female, 12 (66%) were male, and one (6%) identified as non-binary. There were four (22%) T&O year one, six (33%) year two, and eight (44%) year three trainees. Ten (56%) trainees had previous simulation experiences in training, such as VR simulations on the anterior approach to the hip, moulages, and cadaveric simulation. Sixteen (89%) trainees reported that operative numbers were affected by the COVID-19 pandemic, where half of them had seen a 25-50% reduction. Extra operation lists (94%), cadaveric courses (83.3%), and simulation courses (83%) were the most suggested recommendations from the trainees to help bridge the gap in training.

**Table 1:** Trainee demographics and opinion on intervention to bridge the training gap (n=18). T&O: trauma and orthopedics

Questionnaire item	Trainees (%)
Trainee level	
Postgraduate T&O trainee year 1	4 (22%)
Postgraduate T&O trainee year 2	6 (33%)
Postgraduate T&O trainee year 3	8 (44%)
Do you have any experience of simulation in training?	
Yes	10 (56%)
No	8 (44%)
Have your operating numbers been affected by COVID?	
Yes	16 (89%)
No	2 (11%)
If yes to the above, what percentage reduction?	
<25%	7 (39%)
25-50%	9 (50%)
50-75%	1 (6%)
>75%	1 (6%)
What would help bridge the gap in training?	
Extra operating lists	17 (94%)
Cadaveric courses	15 (83%)
Simulation courses	15 (83%)
Regular access to surgical simulators	12 (67%)
Extending the length of training	4 (22%)

Preparing for a new or unfamiliar operation

**Figure 1:**
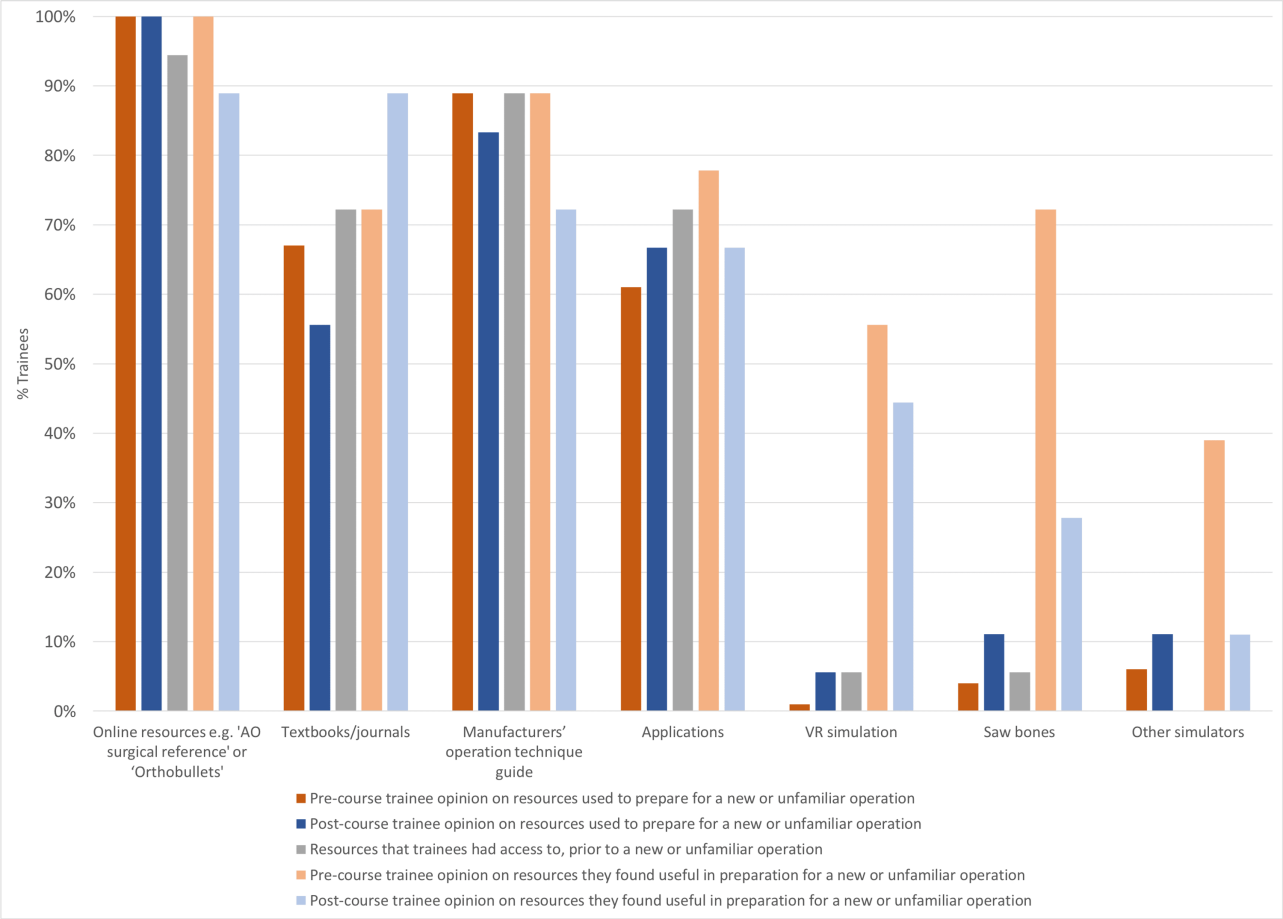
Trainee opinion on resources for preparing a new or unfamiliar operation. AO: Arbeitsgemeinschaft für Osteosynthesefragen

Trainee opinions on preparing for a new or unfamiliar operation before and after course completion were summarized in Table 2 and Figure 1. Trainee access to resources for a new or unfamiliar operation was summarized in Table 3. Online resources, manufacturers’ operation technique guides, and textbooks or journals were consistently the most popular resources for preparing a new or unfamiliar operation, were highly accessible, and were deemed useful to trainees. The lesser-used medium to high-fidelity resources, such as Sawbones and VR simulation, had low accessibility to trainees and saw little increase in trainees likely to use them after the course. Although trainees found them moderately to strongly useful before the course, their perceived utility decreased after the course. There was no significant difference in opinion regarding the resources being used to prepare for a new or unfamiliar operation and the resources that trainees deemed useful to prepare for a new or unfamiliar operation, except for a significant decrease in perceived utility of Sawbones (72% vs. 28%, p=0.005). Only one (6%) trainee used VR simulation for preparation. All trainees had none to minimal access to surgical simulators to prepare for unfamiliar operations, with two-thirds (68%) having no access at all.

**Table 2:** Trainees’ preparation for a new or unfamiliar operation before and after the course (n=18). AO: Arbeitsgemeinschaft für Osteosynthesefragen; VR: virtual reality

Questionnaire item	Pre-course trainee opinion (%)	Post-course trainee opinion (%)	p- Value
What resources do you use to prepare for a new or unfamiliar operation?			
Online resources, e.g., “AO surgical reference” or “Orthobullets”	18 (100%)	18 (100%)	1.000
Textbooks/journals	12 (67%)	10 (56%)	0.317
Manufacturers’ operation technique guide	15 (89%)	15 (83%)	1.000
Applications	11 (61%)	12 (67%)	0.705
VR simulation	1 (6%)	1 (6%)	1.000
Sawbones	7 (39%)	2 (11%)	0.059
Other simulators	1 (6%)	2 (11%)	0.564
Which of these resources are useful in preparation for a new or unfamiliar operation?			
Online resources, e.g., “AO surgical reference” or “Orthobullets”	18 (100%)	16 (89%)	0.157
Textbooks/journals	13 (72%)	16 (89%)	0.180
Manufacturers’ operation technique guide	16 (89%)	13 (72%)	0.083
Applications	14 (78%)	12 (67%)	0.317
VR simulation	10 (56%)	8 (44%)	0.414
Sawbones	13 (72%)	5 (28%)	0.005
Other simulators	5 (39%)	2 (11%)	0.257

**Table 3:** Trainees’ access to resources in preparation for a new or unfamiliar operation (n=18). AO: Arbeitsgemeinschaft für Osteosynthesefragen; VR: virtual reality

Questionnaire item	Trainees (%)
How much access have you had to a surgical simulator to prepare for unfamiliar operations?	
None	12 (67%)
Minimal	6 (33%)
On request	0 (0%)
Full, open access	0 (0%)
Which of these resources do you have access to, prior to a new or unfamiliar operation?	
Online resources, e.g., “AO surgical reference” or “Orthobullets”	17 (94%)
Textbooks/journals	13 (72%)
Manufacturers’ operation technique guide	16 (89%)
Applications	13 (72%)
VR simulation	1 (6%)
Sawbones	1 (6%)
Other simulators	0 (0%)
How much access have you had to a surgical simulator to prepare for unfamiliar operations?	
None	12 (67%)
Minimal	6 (33%)
On request	0 (0%)
Full, open access	0 (0%)

Significance of surgical simulation

**Figure 2:**
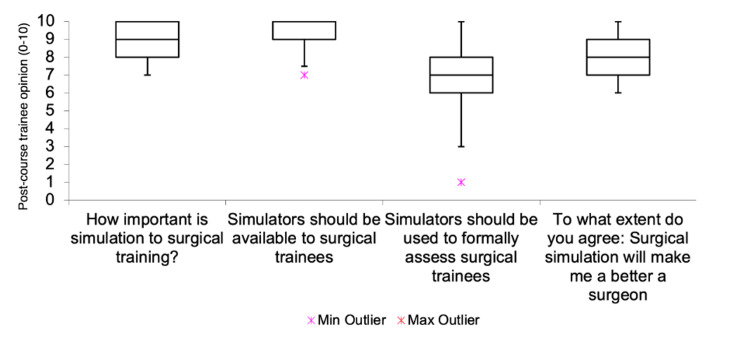
Trainee opinion on the importance of surgical simulation (n=18). A 10-point Likert scale was used, where a score of 1 indicated disagreement or a lack of importance, and a score of 10 indicated agreement or importance.

The opinions of trainees on the importance of surgical simulation before and after course completion were visualized in Figure 2 and Table 4. There was an overall increase in agreement in all questionnaire items on this topic after course completion. The trainees strongly agreed that simulators should be made more available to them (pre- vs. post-course median score: 9 vs. 10, p=0.061), and surgical simulation would make them better surgeons (7 vs. 8, p=0.078). There was a significant increase in agreement between trainees strongly agreeing that simulation was important to surgical training (7 vs. 9, p=0.011) and simulators should be used to formally assess them (4 vs. 8, p=0.006) after the course. Simulators being utilized in assessment had the widest ranges with outliers, which may be due to the unfamiliarity of relying on simulation to demonstrate competency and confidence in supervising attendings. However, the course demonstrably highlighted the usefulness of simulation exercises in assessing a trainee as safe prior to performing the procedure on a patient, thus prioritizing patient safety.

**Table 4:** Trainee opinion on the importance of surgery before and after the course (n=18). P-values <0.05 considered statistically significant. A 10-point Likert scale was used where a score of 1 showed disagreement or a lack of importance, and a score of 10 showed agreement or importance.

Questionnaire item	Median pre-course trainee opinion±SD	Median post-course trainee opinion±SD	Median difference	p-Value
How important is simulation to surgical training?	7±1.86	9±1.18	2	0.011
Simulators should be available to surgical trainees	9±1.72	10±1.07	1	0.061
Simulators should be used to formally assess surgical trainees	4±2.66	7±2.38	3	0.006
To what extent do you agree: surgical simulation will make me a better surgeon	7±1.50	8±1.18	1	0.078

Opinions on orthopedic simulation

Pre-course and post-course opinions of trainees on orthopedic simulation are summarized in Table 5. The trainees consistently agreed that orthopedic simulation should be used in open access simulation labs (pre-course vs. post-course opinion: 100% vs. 83%), regional training days (83% vs. 83%), and as home kits, such as VR headsets and arthroscopy boxes (83% vs. 72%).

**Table 5:** Trainee opinion on orthopedic simulation before and after the course (n=18). AO: Arbeitsgemeinschaft für Osteosynthesefragen; VR: virtual reality.

Questionnaire item	Pre-course trainee opinion (%)	Post-course trainee opinion (%)	p-Value
How should orthopedic simulation be used?			
Home kits, e.g., VR headset, arthroscopy boxes	15 (83%)	13 (72%)	0.480
Open-access simulation labs	18 (100%)	15 (83%)	0.083
Regional training days	15 (83%)	15 (83%)	1.000
Aptitude testing for surgical training	6 (33%)	8 (44%)	0.414
Formal assessments	3 (17%)	4 (22%)	0.655
What in your opinion are the positive elements of surgical simulation?			
Safe practice for surgical procedures	17 (94%)	17 (94%)	1.000
Improve technical skills	15 (83%)	16 (89%)	0.655
Enjoyment	10 (56%)	13 (72%)	0.317
No time or service pressures	14 (78%)	10 (56%)	0.102
What in your opinion are the negative elements of surgical simulation?			
Not useful	0 (0 %)	0 (0%)	1.000
Unrealistic	6 (33%)	5 (28%)	0.564
Other methods are more effective	4 (22%)	2 (11%)	0.157
Limited access	14 (78%)	15 (83%)	0.655
Lack of senior guidance/instruction	7 (39%)	5 (28%)	0.317

The surgical simulation was consistently praised for promoting safe practice for surgical procedures (94% for both). Trainees increasingly agreed that surgical simulation improved technical skills (83% vs. 89%). They found it more enjoyable (56% vs. 72%) but agreed less on training having no time or service pressures (78% vs. 56%).

None of the trainees reported that surgical simulation was not useful. The major criticisms of the surgical simulation were its limited access, especially to high-fidelity modalities (78% vs. 83%), lack of senior guidance or instructions (39% vs. 28%), and being unrealistic (33% vs. 28%). There was no significant difference in trainee opinion regarding negative opinions of simulation pre-course and post-course. Trainees consistently rated a strongly positive opinion on all tasks in the course involving all levels of fidelities, with a median score of 8/10.

The outcome of null hypothesis

The null hypothesis was not accepted. There was a significant increase in both agreement and consensus that simulation is important to surgical training and to be accepted in formal assessments. Hence, it is concluded that there was a difference in the educational value after exposure to various simulation modalities of varying fidelity.

## Discussion

Principal findings

Our study investigated surgical simulation training experiences among T&O trainees amid the COVID-19 pandemic. Trainees strongly emphasized the importance of simulation training, the necessity for its ready accessibility, its role in formal assessment, and being able to become a better surgeon from it. The null hypothesis was not accepted. Trainees strongly favored simulation training of all degrees of fidelity, but would only utilize readily available low-fidelity resources when preparing for a new or unfamiliar operation, even if medium to high-fidelity simulation was available.

Accessibility to simulation modalities

There was a decrease in operative numbers by ≤25% for 88% of the trainees, consistent with other specialties [2]. Only 6% of the trainees had access to VR simulation to prepare for new or unfamiliar operations, potentially due to limited availability [16]. Therefore, it could be beneficial to fund a skills laboratory for VR headsets or simulators available to trainees for practicing key procedures. All trainees recognized the value of simulation training in surgery and its benefits in terms of safety and technical skill acquisition post-course (Table 4).

Limitations of current simulation solutions

Trainees found it useful for learning procedural steps prior to operating in reality, particularly for unfamiliar procedures but complained that the simulators in the course were awkward to use, lacked haptic feedback, and required senior supervision for effective use. The VR simulators were originally designed to showcase implant products, thus potentially neglecting trainees' educational needs applicable to other implants. The identification and assessment of errors made by trainees in simulation, similar to scoring systems in video games, could be used to achieve better performance.

High-fidelity simulators could address these limitations but are associated with high costs and limited by the manufacturers’ software development, exacerbating their already minimal availability [17]. VR simulation is underused in T&O compared to other surgical specialties, due to associated logistics, and the lack of a mandatory and dedicated training time [23,24]. Addressing these issues could bridge the gap in the implementation of simulation training.

Acceptability of simulation in formal training

There was a significant increase (4 vs. 7, p=0.006) in trainees post-course who strongly advocated for formal assessment through orthopedic simulation. There is a need for a universal and validated consensus on outcome objectives for such assessments [25]. Polce et al. highlighted the necessity of standardizing outcome measures for available orthopedic simulators and heterogeneous training programs [26]. Expert trainers need to define the skills and procedures to be taught, as well as consider the opinions of trainees, who will ultimately benefit from such teaching. These elements are crucial in shaping the syllabus to maximize skill acquisition, achieve competency, and ensure safe performance in the operating theater [27]. VR training may help develop a safe, trainee-friendly environment where mistakes can be made without risking patient safety, provide personalized feedback, and allow surgical skills to be refined until proficiency is achieved [28].

Contrast and comparison with pre-existing literature

This study's findings on T&O trainee opinions were consistent with other studies regarding the lack of access to high-fidelity simulators and their preference for using highly assessable resources such as online resources and textbooks when preparing for a new or unfamiliar operation [16,29]. Seil et al. demonstrated almost all T&O trainees from Switzerland, France, Germany, and Luxembourg found simulation training useful and were in overwhelming favor of mandatory simulation training [22]. The trainees in this study and the European trainees investigated by Seil et al. mostly demonstrated the perceived importance of simulation training [22]. However, trainee perceptions of learning an intervention were deemed to have little value on learning outcomes in the context of self-assessment, where “students don’t know what they know” [30,31]. Failing to address issues like accessibility and exposure to simulation, which is an objective measure, may be detrimental to the training of T&O trainees.

Strengths of study

Our study design minimized recall biases and reduced the reliance on unsubstantiated information by immediately collecting trainee opinions after the completion of the course. The use of a validated questionnaire ensured that its content validity was adequate enough to assess trainee opinion. Multiple simulation modalities of varying fidelities were utilized to showcase possibilities for access and use of educational technologies. This study did not suffer from selection, attrition, and recall biases.

Limitations and future directions

The limitations of this study included a small sample size that may not be representative of the general T&O trainee population. Future studies will incorporate standardized objective assessment of the simulation station or proof of training effect/decay which was otherwise outside the scope of this study. Trainees emphasized that while simulation training would be a productive addition to the surgical curriculum, such simulation could not replace actual operating numbers [22]. Hence simulation is never meant to displace proctorship but to simply be used as an adjunct to enhance the educational impact through practice and reflection in a safe environment to demonstrate competency and confidence without compromising patient safety.

## Conclusions

This study highlighted the significance of simulation training, and of varying fidelities, in complementing operative experience in the training of junior postgraduate T&O trainees. However, limited resource accessibility remains a significant concern, as previously noted in the literature. The implementation gap of simulation training in T&O should be addressed to ensure that trainees can fully benefit from these resources.

Moreover, T&O trainees recognized the importance of simulation training in their surgical education, advocated incorporating it into formal assessments, and considered that it should be more integrated into their curricula. Integrating simulation training in T&O training programs has the potential to maximize skills acquisition, help trainees achieve competency, and ensure safe practice in the operating theatre. Future efforts should focus on providing access to high-fidelity simulators and VR headsets, identifying and addressing the logistical challenges, and developing universal and validated consensus on outcome objectives for assessments.

## Appendices

**Table 6:** Pre-course questionnaire and participants' views. T&O: trauma and orthopedics; AO: Arbeitsgemeinschaft für Osteosynthesefragen

Pre-course questionnaire
Full name
Short-answer text input.
What level of training are you?
Choose one of the following: pre-T&O training, T&O training year 1, T&O training year 2, T&O training year 3, T&O training year 4, T&O training year 5, T&O training year 6, trust post, or other.
Do you have any experience of simulation in training?
Choose one of the following: Yes, No.
If yes - what experience do you have?
Short-answer text input.
Have your operating numbers been affected by COVID?
Choose one of the following: Yes, No.
If yes to the above, what percentage reduction?
Choose one of the following: <25%, 25-50%, 50-75%, >75%.
What would help bridge the gap in training?
Choose choice(s) applicable: extra operating lists, cadaveric courses, simulation courses, regular access to surgical simulators, and extending the length of training.
How important is simulation to surgical training?
Rate 1-10 (1=disagree, 10=agree)
Simulators should be available to surgical trainees.
Rate 1-10 (1=disagree, 10=agree)
How should orthopedic simulation be used?
Choose choice(s) applicable: home kits, e.g., VR headset, arthroscopy boxes; open access simulation labs; regional training days; aptitude testing for surgical training; formal assessments.
What resources do you use to prepare for a new or unfamiliar operation?
Choose choice(s) applicable: online resources, e.g., “AO surgical reference” or “Orthobullets,” textbooks/journals, manufacturers’ operation technique guide, applications, VR simulation, Sawbones, and other simulators.
Which of these resources do you have access to, prior to a new or unfamiliar operation?
Choose choice(s) applicable: online resources, e.g., “AO surgical reference” or “Orthobullets,” textbooks/journals, manufacturers’ operation technique guide, applications, VR simulation, Sawbones, and other simulators.
Which of these resources are useful in preparation for a new or unfamiliar operation?
Choose choice(s) applicable: online resources, e.g., “AO surgical reference” or “Orthobullets,” textbooks/journals, manufacturers’ operation technique guide, applications, VR simulation, Sawbones, and other simulators.
Simulators should be used to formally assess surgical trainees.
Rate 1-10 (1=disagree, 10=agree)
To what extent do you agree that surgical simulation will make me a better surgeon?
Rate 1-10 (1=disagree, 10=agree)
How much access have you had to a surgical simulator to prepare for unfamiliar operations?
Choose choice(s) applicable: none, minimal, on request, full, open access.
What in your opinion are the positive elements of surgical simulation?
Choose choice(s) applicable: safe practice for surgical procedures, improved technical skills, enjoyment, no time or service pressures.
What in your opinion are the negative elements of surgical simulation?
Choose choice(s) applicable: not useful, unrealistic, other methods are more effective, limited access, lack of senior guidance/instruction.

**Table 7:** Post-course questionnaire and participants' views. T&O: trauma and orthopedics; AO: Arbeitsgemeinschaft für Osteosynthesefragen

Post-course questionnaire
Full name
Short-answer text input.
What level of training are you?
Choose one of the following: pre-T&O training, T&O training year 1, T&O training year 2, T&O training year 3, T&O training year 4, T&O training year 5, T&O training year 6, trust post, and other.
Please rate: VR total knee replacement
Rate 1-10 (1=poor, 10=excellent). Additional comment here.
Please rate: VR femoral nailing
Rate 1-10 (1=poor, 10=excellent). Additional comment here.
Please rate: Fundamentals of Arthroscopic Surgery Training Program
Rate 1-10 (1=poor, 10=excellent). Additional comment here.
Please rate: Sawbones femoral nailing
Rate 1-10 (1=poor, 10=excellent). Additional comment here.
How important is simulation to surgical training?
Rate 1-10 (1=poor, 10=excellent)
Simulators should be available to surgical trainees.
Rate 1-10 (1=poor, 10=excellent)
How should orthopedic simulation be used?
Choose choice(s) applicable: home kits, e.g., VR headset, arthroscopy boxes; open access simulation labs; regional training days; aptitude testing for surgical training; formal assessments.
What resources do you use to prepare for a new or unfamiliar operation?
Choose choice(s) applicable: online resources, e.g., “AO surgical reference” or “Orthobullets,” textbooks/journals, manufacturers’ operation technique guide, applications, VR simulation, Sawbones, and other simulators.
Which of these resources do you have access to, prior to a new or unfamiliar operation?
Choose choice(s) applicable: online resources, e.g., “AO surgical reference” or “Orthobullets,” textbooks/journals, manufacturers’ operation technique guide, applications, VR simulation, Sawbones, and other simulators.
Which of these resources are useful in preparation for a new or unfamiliar operation?
Choose choice(s) applicable: online resources, e.g., “AO surgical reference” or “Orthobullets,” textbooks/journals, manufacturers’ operation technique guide, applications, VR simulation, Sawbones, and other simulators.
Simulators should be used to formally assess surgical trainees.
Rate 1-10 (1=disagree, 10=agree)
To what extent do you agree that surgical simulation will make me a better surgeon?
Rate 1-10 (1=disagree, 10=agree)
How much access have you had to a surgical simulator to prepare for unfamiliar operations?
Choose choice(s) applicable: none, minimal, on request, full, open access.
What in your opinion are the positive elements of surgical simulation?
Choose choice(s) applicable: safe practice for surgical procedures, improved technical skills, enjoyment, no time or service pressures.
What in your opinion are the negative elements of surgical simulation?
Choose choice(s) applicable: not useful, unrealistic, other methods are more effective, limited access, lack of senior guidance/instruction.
